# Short-Term Outcomes in Planned Versus Unplanned Surgery for Spinal Metastases

**DOI:** 10.3390/cancers17142403

**Published:** 2025-07-20

**Authors:** Ali Haider Bangash, Sertac Kirnaz, Rose Fluss, Victoria Cao, Alexander Alexandrov, Liza Belman, Yaroslav Gelfand, Saikiran G. Murthy, Reza Yassari, Rafael De la Garza Ramos

**Affiliations:** 1 Spine Tumor Mechanics and Outcomes Research (TUMOR) Lab, Montefiore Medical Center, Albert Einstein College of Medicine, Bronx, NY 10467, USA; alhaider@montefiore.org (A.H.B.); skirnaz@montefiore.org (S.K.); abbani@montefiore.org (R.F.); victoria.cao@einsteinmed.edu (V.C.); alexander.alexandrov@einsteinmed.edu (A.A.); liza.belman@einsteinmed.edu (L.B.); ygelfand@montefiore.org (Y.G.); samurthy@montefiore.org (S.G.M.); ryassari@montefiore.org (R.Y.); 2 Department of Neurosurgery, Montefiore Medical Center, Albert Einstein College of Medicine, Bronx, NY 10467, USA

**Keywords:** metastatic spine disease, unplanned surgery, Clavien–Dindo, failure to rescue, mortality, length of stay

## Abstract

**Background/Objectives:** Metastatic spine disease (MSD) affects a significant proportion of patients with advanced malignancies and often necessitates surgical intervention to preserve neurological function, alleviate pain, and maintain spinal stability. While oncologic spine surgery is ideally performed in a planned, semi-elective setting, a substantial number of patients require unplanned (urgent or emergent) surgery due to acute deterioration. The impact of surgical planning status on postoperative outcomes following metastatic spine tumor surgery remains underexplored. This study aimed to compare the patient characteristics and short-term outcomes of those undergoing planned versus unplanned surgery for spinal metastases. **Methods:** We conducted a retrospective cohort study using the American College of Surgeons National Surgical Quality Improvement Program (ACS-NSQIP) database from 2018 to 2023. Patients with disseminated cancer undergoing tumor surgery were identified. Case types were grouped into planned (elective) and unplanned (urgent or emergent). The primary endpoint was failure to rescue (FTR); secondary endpoints included 30-day major complications, 30-day mortality, and length of hospital stay. Univariable and multivariable regression analyses were performed. **Results:** A total of 2147 patients met our inclusion criteria, out of whom 60% (*n* = 1284) underwent planned and 40% (*n* = 863) underwent unplanned surgery. Patients in the unplanned surgery group had a significantly higher prevalence of severe hypoalbuminemia, severe anemia, and ASA class IV status (*p* ≤ 0.001 for all). For our primary endpoint, a multivariable analysis showed a significant association between unplanned surgery and FTR (OR 2.11 [95% CI 1.24 to 3.56]; *p* = 0.005). Significant associations were also found with 30-day mortality (OR 1.84 [95% CI 1.25 to 2.72]; *p* = 0.002) and length of hospital stay (β 2.7 [95% CI 1.97 to 3.43]; *p* < 0.001). However, unplanned surgery could not independently predict 30-day major complications (OR 1.21 [95% CI 0.97 to 1.51]; *p* = 0.08). **Conclusions:** Our study found that unplanned surgery for spinal metastases was associated with significantly higher rates of FTR, 30-day mortality, and extended hospital stay, independent of other covariates. These findings highlight the importance of the timely identification of patients requiring surgery and the potential benefits of semi-elective care.

## Simple Summary

When cancer spreads to the spine, surgery is often needed to preserve mobility, reduce pain, and maintain spinal stability. Ideally, these operations are planned in advance, allowing doctors to prepare patients properly. However, many patients require urgent, unplanned surgery due to sudden worsening of symptoms. We compared outcomes between these two scenarios using data from over 2000 patients across multiple hospitals. Our research found that patients undergoing unplanned spine surgery had significantly higher failure to rescue and complication rates, greater risk of death within 30 days, and longer hospital stays compared to those undergoing planned procedures. These findings highlight the importance of early identification of patients who might need surgical intervention and suggest that, whenever possible, allowing time for proper preparation before surgery may lead to better outcomes. This knowledge can help improve care pathways for cancer patients with spine involvement and potentially save lives.

## 1. Introduction

Metastatic spine disease (MSD) is an important clinical challenge as it affects up to 60% of patients with advanced cancer [1]. The spine is reported to be the most common site of skeletal metastases, with thoracic spine involvement in approximately 70% of cases, followed by the lumbar (20%) and cervical regions (10%) [2]. These lesions can cause debilitating complications including intractable pain, neurological deficits, and pathological fractures, substantially diminishing patients’ quality of life and functional independence [3]. Furthermore, the management of MSD places a considerable burden on healthcare systems, with increasing incidence as cancer survival rates improve and diagnostic capabilities advance [4].

Surgical intervention for MSD has evolved considerably over recent decades, with current approaches involving minimally invasive techniques and enabling technologies [4]. The decision to operate is guided by several factors, including the patient’s overall prognosis, neurological status, spinal stability, and tumor histology [5]. While many of these procedures are performed electively after thorough multidisciplinary evaluation and preoperative optimization, a substantial proportion occurs in an unplanned (urgent or emergent) setting, particularly in cases of acute neurological deterioration or spinal instability with impending cord compression [6].

Despite the extensive literature examining outcomes following surgery for spinal metastases, the specific impact of surgical planning status on postoperative outcomes remains incompletely characterized. Previous studies have primarily focused on factors such as primary tumor histology, preoperative functional status, and extent of disease as predictors of surgical outcomes [7,8]. Upon recognizing that the distinction between planned and unplanned interventions may represent a critical, potentially modifiable factor influencing postoperative morbidity and mortality, an attempt has been made to explore its impact on surgical outcomes [9]. Furthermore, such studies have mostly been based on data from single institutions [10,11,12]. Therefore, while urgency of intervention has been established as a predictor of adverse outcomes across other spine surgical pathologies such as deformity [13], degeneration [14], and trauma [15], its specific influence in the context of surgery for spinal metastases deserves further investigation.

The distinction between planned and unplanned surgery is particularly critical in the context of MSD due to several important physiological considerations. Patients undergoing planned procedures benefit from comprehensive preoperative optimization, including the correction of nutritional deficiencies, management of anemia, cardiopulmonary conditioning, and detailed anesthetic planning [16,17,18]. In contrast, those requiring unplanned intervention due to acute neurological deterioration or impending spinal instability typically lack these advantages, potentially entering surgery with uncorrected hypoalbuminemia, significant anemia, fluid and electrolyte imbalances, and suboptimal cardiopulmonary status [19,20,21]. We hypothesize that these differences in preoperative physiological status represent primary drivers of the disparate outcomes observed between planned and unplanned surgical interventions for spinal metastases. Furthermore, the psychological preparation of patients and coordination of multidisciplinary care teams may be compromised in urgent scenarios, potentially contributing to suboptimal perioperative management [22].

Thus, the present study aimed to compare the patient characteristics and outcomes of those undergoing planned versus unplanned surgery for spinal metastases by analyzing a large, multi-institutional, and contemporary cohort from the American College of Surgeons National Surgical Quality Improvement Program (ACS-NSQIP) database.

## 2. Materials and Methods

This study was deemed exempt from review by our institutional review board (2016-6862). This study was a review of publicly available data within the ACS-NSQIP database for the years 2018 to 2023.

The inclusion criteria were as follows: (1) patients with a diagnosis of “disseminated cancer” as collected by the ACS-NSQIP; (2) patients managed with surgery for spinal metastases (identified via CPT codes; Supplementary Table S1); (3) patients not dependent on ventilator; and (4) patients not having an American Society of Anesthesiologists (ASA) class 5.

**Figure 1:**
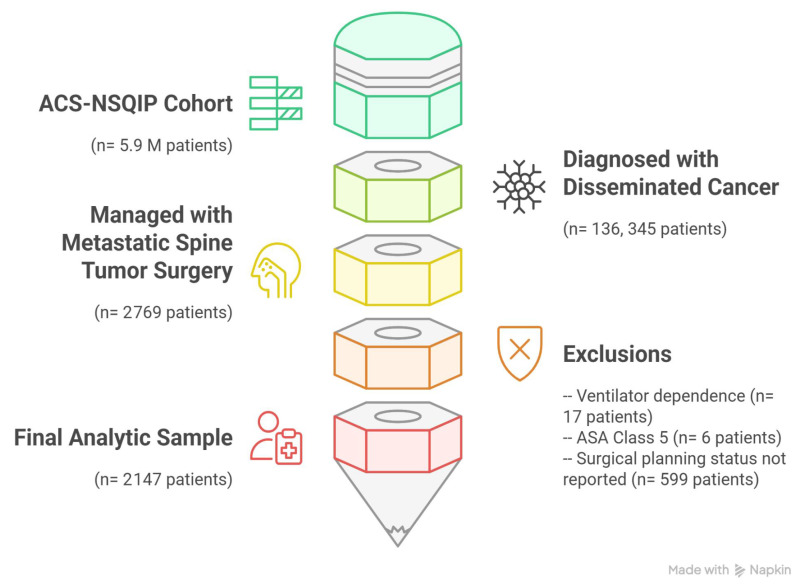
Patient inclusion flowchart for 2147 patients managed with metastatic spine tumor surgery for disseminated cancer extracted from ACS-NSQIP 2018–2023 database (this figure was developed using the Napkin AI web application [Corresponding website address: https://www.napkin.ai/] [Accessed date: 8 May 2025]).

From an initial cohort of 5,989,984 records, only 2% had a diagnosis of disseminated cancer (136,345 of 5,989,984). Within this disseminated cancer cohort, 2% of patients were managed with surgery for spinal metastases (2769 of 136,345). Seventeen patients were excluded because they were dependent on a ventilator, whereas six patients were excluded because they had ASA class 5. Moreover, 599 patients were excluded since their surgical planning status was not reported, leaving 2147 patients in the final analytic sample (Figure 1).

### 2.1. Collected Variables

Collected patient data included age at surgery, sex, preoperative functional status, body mass index (BMI), and history of chronic obstructive pulmonary disease (COPD), congestive heart failure (CHF), and bleeding disorders. Data on preoperative dialysis, modified Frailty Index—5 (mFI5), ASA class, history of chronic steroid use, preoperative albumin level, hematocrit (HCT) level, and white cell count (WCC) were also extracted. Mild hypoalbuminemia was defined as an albumin level between 2.5 and 3.5 g/dL, whereas severe hypoalbuminemia was defined as an albumin level ≤2.5 g/dL [23]. Furthermore, cut-offs for mild anemia (HCT: 35.1–39.0%), moderate anemia (HCT: 30.1–35.0%), and severe anemia (HCT: ≤ 30.0%) were defined [24].

The collected operative variables included planned (elective) vs. unplanned (urgent/emergent) surgery, operative time, fusion procedure (vs. decompression only), corpectomy, multilevel corpectomy, and perioperative transfusion.

### 2.2. Endpoints

The primary endpoint was failure to rescue (FTR), defined as the occurrence of major (Clavien–Dindo Grade 3 or 4) complication(s) plus mortality within 30 days of the index operation. The secondary endpoints included 30-day major complication(s), 30-day mortality, and length of hospital stay. Major complications included any of the following adverse events: deep surgical site infection, organ space surgical site infection, unplanned reintubation, prolonged ventilation for ≥48 h, pulmonary embolism, a cerebrovascular accident, renal failure, myocardial infarction, cardiac arrest, sepsis, septic shock, pneumonia, or unplanned reoperation.

### 2.3. Statistical Analysis

Exploratory data analysis was first performed. The categorical variables were expressed as percentages of the total and continuous variables were expressed as mean (with standard deviation). Categorical variables among unplanned and planned surgery cohorts were compared via Pearson’s chi-square test of association, whereas the independent-sample *t*-test was implemented to compare continuous variables among cohorts. Univariable regression analyses were undertaken to explore the association of each independent variable with primary and secondary endpoints. The primary independent variable was planned (elective) vs. unplanned (urgent or emergent) surgery. Multivariable regression analyses were undertaken to determine the independent predictors of primary and secondary endpoints. Only factors with a *p*-value < 0.05 in univariable analysis were included in the multivariable regression analyses to reduce the likelihood of overfitting given the relatively low number of events. Statistical analyses were performed using IBM SPSS (version 26), Stata/IC (version 15), and GraphPad Prism (version 10). Statistical significance was defined as a *p*-value < 0.05.

## 3. Results

This study reported on a total of 2147 patients with a mean age of 62 (±12.5) years and a 60% (*n* = 1287) male population (Table 1). From the total cohort, 1284 underwent planned surgery (60%) and 863 underwent unplanned surgery (40%). Patients in the unplanned surgery group were more likely to be male (*p* = 0.042), have a history of a bleeding disorder (*p* = 0.002), and have a severe life-threatening systemic disease (ASA class 4) (*p* < 0.001), severe hypoalbuminemia (*p* < 0.001), and severe anemia (*p* < 0.001) compared to patients in the planned surgery group.

Operative characteristics are summarized in Table 1. Patients in the unplanned surgery group had a shorter operative time (*p* < 0.001) and were significantly less likely to undergo corpectomy procedures (*p* < 0.001).

### 3.1. Failure to Rescue

**Figure 2:**
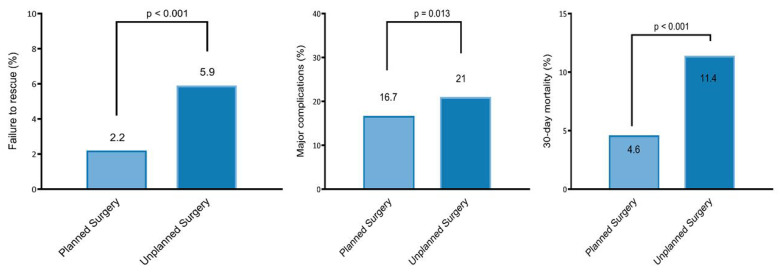
Failure to rescue (**left**), 30-day major complications (**middle**), and mortality rate (**right**) in the planned versus unplanned surgery groups.

In the 30-day postoperative period, 4% of patients (79 of 2147) experienced FTR—the rate was 2.2% versus 5.9% in the planned versus unplanned groups, respectively (*p* < 0.001) (Figure 2). The univariable and multivariable analyses are summarized in Supplementary Table S2 and Table 2, respectively. After controlling for preoperative functional health status, history of CHF, history of a bleeding disorder, mFI-5, ASA, preoperative albumin level, preoperative HCT level, preoperative WCC, and perioperative transfusion, unplanned surgery was found to be significantly associated with FTR (OR 2.11 [95% CI 1.24 to 3.56]; *p* = 0.005) in our multivariable model (Table 2).

### 3.2. Thirty-Day Major Complications

The major complication rate was 18% (396 of 2147)—16.7% versus 21% in the planned versus unplanned groups, respectively (*p* = 0.013) (Figure 2). The univariable and multivariable analyses are summarized in Supplementary Table S3 and Table 3, respectively. When controlling for preoperative functional health status, history of CHF, mFI-5, ASA, preoperative albumin level, preoperative HCT level, preoperative WCC, multilevel corpectomy, and perioperative transfusion, unplanned surgery was found not to independently predict 30-day major complications (OR 1.09 [95% CI 0.84 to 1.41]; *p* = 0.49) (Table 3).

### 3.3. Thirty-Day Mortality

The overall 30-day mortality rate was 7% (157 of 2147)—4.6% versus 11.4% in the planned versus unplanned groups, respectively (*p* < 0.001) (Figure 2). The univariable and multivariable analyses are summarized in Supplementary Table S4 and Table 4, respectively. When controlling for age, male sex, preoperative functional health status, history of CHF, history of a bleeding disorder, mFI-5, ASA, preoperative albumin level, preoperative HCT level, preoperative WCC, operative time, fusion procedure (vs. decompression only), and perioperative transfusion, unplanned surgery (OR 1.84 [95% CI 1.25 to 2.72]; *p* = 0.002) was found to independently predict 30-day mortality (Table 4).

### 3.4. Length of Hospital Stay

The mean hospital length of stay was 10 (± 7.5) days—9 days ± 7 days vs. 12 ± 7 days in the planned versus unplanned groups, respectively (*p* < 0.001). The univariable and multivariable analyses are summarized in Supplementary Table S5 and Table 5, respectively. When controlling for preoperative functional health status, history of a bleeding disorder, preoperative dialysis, mFI-5, ASA, preoperative albumin level, preoperative HCT level, preoperative WCC, operative time, fusion procedure (vs. decompression only), and perioperative transfusion, unplanned surgery (β 2.7 [95% CI 1.97 to 3.43]; *p* < 0.001) was found to independently predict length of hospital stay (Table 5).

## 4. Discussion

In our current study of patients undergoing planned versus unplanned surgery for spinal metastases, we found that patients undergoing unplanned surgery had a significantly higher prevalence of severe hypoalbuminemia, severe anemia, and ASA class 4 status, suggesting more advanced disease and compromised physiological reserves. This group of patients had a shorter duration of surgery and were less likely to receive vertebral corpectomy. When comparing outcomes between groups in a multivariable model, we found that unplanned surgery independently predicted FTR, 30-day mortality, and length of stay.

Our findings align with previous studies examining the impact of surgical urgency on outcomes for patients undergoing surgery for spinal metastases. Zeoli T et al. reported that patients managed in an emergent setting had a significantly higher comorbidity burden and experienced significantly longer length of hospital stay and shorter survival [11]. However, in contrast to our findings, patients managed in an emergent setting in that study were significantly more likely to undergo more complex surgical interventions, with as an increased number of spinal levels decompressed and higher costotransversectomy rates [11]. Kanda Y et al. also reported significantly shorter survival for patients managed with emergency surgery, although in contrast to our findings, they reported significantly longer operative time and no impact on postoperative complications [10]. However, to the best of our knowledge, our study is the first attempt to explore unplanned oncologic surgery as a predictor of FTR for patients managed for spinal metastases. In the broader oncologic surgery literature, unplanned operations have consistently demonstrated inferior outcomes with significantly higher morbidity and mortality [25,26,27].

Unfortunately, unplanned surgery remains a frequent occurrence in patients with spinal metastases, particularly in cases of acute neurological deterioration [28]. The decision to proceed with urgent intervention often represents a balance between the risks of surgery and the consequences of delay. Our findings not only second appropriate urgent intervention when indicated, but also highlight the need for enhanced perioperative management strategies for this high-risk population in unplanned surgery settings [29]. When clinically feasible, temporary stabilization measures that allow for preoperative optimization such as percutaneous fenestrated pedicle screws and cement augmentation may be beneficial [30,31]. For inevitably urgent cases, the allocation of appropriate resources, including experienced surgical teams and intensive care support, may help to mitigate risks [32].

Nonetheless, efforts should also be directed towards decreasing the rates of unplanned interventions. Improving referral patterns from primary care providers and medical oncologists to spine surgeons could facilitate the earlier evaluation of patients with spinal metastases [33,34]. The implementation of standardized screening protocols for patients with cancers that commonly metastasize to the spine may help to identify lesions before they become symptomatic or cause neurological compromise [35]. We suggest implementing automatic spine surgery consultation when vertebral lesions are detected on initial staging scans and incorporating the Spinal Instability Neoplastic Score (SINS) as a routine assessment tool during oncology follow-up visits. Additionally, reducing administrative delays in the referral process through streamlined pathways and expedited imaging protocols could allow more patients to undergo thorough preoperative assessment and optimization [33]. Educational initiatives targeting non-spine specialists focused on the early warning signs of spinal instability or impending neurological compromise may also promote timely referrals. Multidisciplinary tumor boards that include spine surgeons could further enhance communication and facilitate proactive surgical planning for high-risk patients [36]. The establishment of dedicated nurse navigators for patients with MSD could further expedite care coordination, while specialized “prehabilitation” programs could optimize nutritional status and correct anemia before surgical intervention becomes urgent. These preventative approaches may ultimately reduce the proportion of patients requiring unplanned surgery and potentially improve overall outcomes [37,38].

Future research should focus on several key areas to build upon these findings. Studies examining the timing continuum, rather than the binary classification of planned versus unplanned surgery, might identify critical windows for intervention that balance urgency with optimization. Moreover, qualitative research exploring decision-making processes in cases managed with unplanned surgery could identify institutional and system-level factors influencing outcomes. In addition to this, the development and validation of risk prediction models specifically for unplanned interventions would help to guide clinical decision-making and resource allocation. Such research should ideally incorporate functional outcomes and quality-of-life measures beyond the traditional morbidity and mortality metrics, providing a more comprehensive understanding of the impact of surgical timing on patient-centered outcomes.

### Limitations

There are several limitations to this study. The retrospective nature of the analysis limited causal inference regarding the relationship between surgical planning status and outcomes. The ACS-NSQIP database, while robust for surgical quality assessment, has inherent constraints including a lack of cancer-specific variables that significantly influence surgical decision-making such as neurological/ambulatory status, primary tumor type, extent of metastatic burden, previous oncologic treatments, and radiation history. These unmeasured confounders may substantially impact both the likelihood of unplanned surgery and subsequent outcomes. We also acknowledge that excluding 599 patients (22% of eligible cases) with a missing surgical planning status may have introduced selection bias. Additionally, while we chose multivariable regression with careful covariate selection to maintain statistical power, propensity score matching could have further balanced groups and addressed residual confounding.

Furthermore, the specific indications for unplanned surgery were not available, limiting our ability to distinguish between potentially modifiable and non-modifiable factors driving urgency. The database also lacks information on institutional factors such as hospital volume, surgeon experience, and availability of multidisciplinary teams, which may influence both the decision for urgent intervention and subsequent outcomes. Regarding generalizability, our findings reflect outcomes from NSQIP-participating institutions, which may have better resources and standardized care protocols than non-participating centers, potentially limiting applicability to all practice settings. Moreover, the 30-day follow-up period may be too short to appreciate the postoperative trajectory of recuperation. In addition to this, while the ACS-NSQIP database captures a wide range of complications, it includes neither functional outcomes nor quality-of-life measures, which are particularly relevant for patients with MSD.

## 5. Conclusions

Our study found that unplanned surgery for spinal metastases was associated with worse 30-day outcomes compared to planned interventions. While our retrospective design cannot establish causality, these suboptimal results likely result from the urgency of the clinical presentation, more advanced patient presentation, and limitations in preoperative optimization. Future research should focus on the earlier identification and enhanced referral of patients with metastatic spine disease, identifying modifiable risk factors within the unplanned surgery pathway, developing targeted preoperative protocols for semi-urgent cases, and investigating the timing continuum to determine optimal windows for intervention that balance clinical urgency with adequate preparation. Prospective, multicenter studies incorporating functional and quality-of-life outcomes alongside traditional morbidity and mortality metrics are particularly needed to validate these findings and better delineate the relationship between surgical planning status and patient outcomes. From a quality improvement perspective, institutions should consider developing and implementing structured care pathways with clear triggers for surgical consultation based on imaging findings or symptom progression.

## Figures and Tables

**Table 1:** Baseline characteristics and operative characteristics of 2147 patients undergoing metastatic spine tumor surgery for disseminated cancer.

Parameters	Planned Surgery	Unplanned Surgery	*p*-Value
Number of patients	1284	863	
Age (mean, SD)	61.87, 12.3	62.81, 12.7	0.08
Male sex (%)	747 (58.2%)	540 (62.6%)	0.042 *
Preoperative functional health status			0.265
Independent (%)	1162 (90.5%)	761 (88.2%)	
Partially dependent (%)	101 (7.9%)	88 (10.2%)	
Totally dependent (%)	16 (1.2%)	12 (1.4%)	
Unknown status (%)	5 (0.4%)	2 (0.2%)	
BMI (mean kg/m^2^, SD)	27.46, 6.1	27.25, 6.4	0.46
History of COPD (%)	50 (3.9%)	41 (4.8%)	0.33
History of congestive heart failure (%)	27 (2.1%)	24 (2.8%)	0.31
History of a bleeding disorder (%)	71 (5.5%)	77 (8.9%)	0.002 *
Preoperative dialysis (%)	4 (0.3%)	4 (0.5%)	0.57
mFI-5 (mean, SD)	0.73, 0.8	0.79, 0.8	0.09
ASA (mean, SD)	3.02, 0.5	3.2, 0.5	<0.001 *
ASA status			<0.001 *
ASA Class 1 (%)	1 (0.1%)	4 (0.5%)	
ASA Class 2 (%)	169 (13.2%)	48 (5.6%)	
ASA Class 3 (%)	912 (71%)	533 (61.8%)	
ASA Class 4 (%)	200 (15.6%)	278 (32.2%)	
Unknown class (%)	2 (0.2%)	0 (0%)	
Chronic steroid use (%)	250 (19.5%)	163 (18.9%)	0.73
Preoperative albumin level (g/dL) (mean, SD)	3.72, 0.6	3.55, 0.6	<0.001 *
Albumin status			<0.001 *
Normoalbuminemia	980 (76.3%)	583 (67.6%)	
Mild hypoalbuminemia	265 (20.6%)	226 (26.2%)	
Severe hypoalbuminemia	39 (3%)	54 (6.3%)	
Preoperative hematocrit level (%) (mean, SD)	36.84, 5.7	36.01, 5.9	0.001 *
Anemic status			0.001 *
Normal	477 (37.1%)	270 (31.3%)	
Mild anemia	354 (27.6%)	221 (25.6%)	
Moderate anemia	280 (21.8%)	211 (24.4%)	
Severe anemia	173 (13.5%)	161 (18.7%)	
Preoperative white cell count (× 10^9^/L) (mean, SD)	8.88, 4.5	9.39, 5	0.016 *
Operative time (hours) (mean, SD)	3.82, 2	3.23, 1.7	<0.001 *
Fusion procedure (%)	874 (68.1%)	494 (57.2%)	<0.001 *
Corpectomy (%)	483 (37.6%)	218 (25.3%)	<0.001 *
Multilevel corpectomy (%)	54 (4.2%)	18 (2.1%)	0.007 *
Perioperative transfusion (%)	246 (19.2%)	161 (18.7%)	0.77

* Statistically significant; SD: standard deviation; BMI: body mass index.

**Table 2:** Multivariable logistic regression of factors associated with failure to rescue for patients managed with metastatic spine tumor surgery for disseminated cancer.

Parameters	Odds Ratio	95% CI	*p*-Value
Male sex	1.39	0.8–2.4	0.23
Preoperative functional health status			
Independent		REFERENCE	
Partially dependent	1.66	0.79–3.48	0.17
Totally dependent	2.97	0.69–12.72	0.14
Unknown status	11.65	1.14–118.13	0.038 ^¶^
History of congestive heart failure	1.96	0.63–6.09	0.24
History of a bleeding disorder	2.06	1.04–4.08	0.037 ^¶^
mFI-5	1.08	0.78–1.5	0.62
ASA	1.39	0.87–2.23	0.16
Preoperative albumin level	0.6	0.39–0.92	<0.019 ^¶^
Preoperative hematocrit level	0.94	0.9–0.99	0.029 ^¶^
Preoperative white cell count	1.07	1.03–1.11	<0.001 ^¶^
Unplanned surgery	2.11	1.24–3.56	0.005 ^¶^
Perioperative transfusion	1.4	0.78–2.5	0.24

^¶^ Independent predictor.

**Table 3:** Multivariable logistic regression of factors associated with 30-day major (Clavien–Dindo Grade 3 or 4) complications for patients managed with metastatic spine tumor surgery for disseminated cancer.

Parameters	Odds Ratio	95% CI	*p*-Value
Preoperative functional health status			
Independent		REFERENCE	
Partially dependent	0.89	0.58–1.38	0.62
Totally dependent	1.2	0.43–3.31	0.72
Unknown status	3.45	0.54–21.92	0.18
History of congestive heart failure	1.58	0.78–3.18	0.2
mFI-5	1.29	1.09–1.52	0.002 ^¶^
ASA	1.13	0.89–1.43	0.28
Preoperative albumin level	0.65	0.53–0.81	<0.001 ^¶^
Preoperative hematocrit level	0.97	0.94–0.99	0.014 ^¶^
Preoperative white cell count	1.02	1–1.05	0.018 ^¶^
Unplanned surgery	1.09	0.84–1.41	0.49
Multilevel corpectomy	1.9	1.06–3.42	0.03 ^¶^
Perioperative transfusion	1.59	1.18–2.13	0.002 ^¶^

^¶^ Independent predictor.

**Table 4:** Multivariable logistic regression of factors associated with 30-day mortality for patients managed with metastatic spine tumor surgery for disseminated cancer.

Parameters	Odds Ratio	95% CI	*p*-Value
Age	1	0.98–1.02	0.56
Male sex	1.34	0.9–2.01	0.14
Preoperative functional health status			
Independent		REFERENCE	
Partially dependent	1.19	0.66–2.16	0.54
Totally dependent	1.74	0.49–6.19	0.39
Unknown status	4.03	0.37–43.46	0.25
History of congestive heart failure	1.68	0.66–4.23	0.27
History of a bleeding disorder	2.29	1.37–3.85	0.002 ^¶^
mFI-5	1.08	0.83–1.39	0.54
ASA	1.07	0.75–1.52	0.68
Preoperative albumin level	0.56	0.4–0.76	<0.001 ^¶^
Preoperative hematocrit level	0.95	0.92–0.99	0.022 ^¶^
Preoperative white cell count	1.08	1.05–1.12	<0.001 ^¶^
Unplanned surgery	1.84	1.25–2.72	0.002 ^¶^
Operative time	0.91	0.8–1.02	0.127
Fusion procedure	0.64	0.42–0.99	0.046 ^¶^
Perioperative transfusion	1.45	0.92–2.27	0.1

^¶^ Independent predictor.

**Table 5:** Multivariable linear regression of factors associated with the length of hospital stay for patients managed with metastatic spine tumor surgery for disseminated cancer.

Parameters	β	95% CI	*p*-Value
Preoperative functional health status			
Independent		REFERENCE	
Partially dependent	1.01	−0.29–2.31	0.12
Totally dependent	3.57	0.1–7.04	0.044 ^¶^
Unknown status	6.07	−0.12–12.27	0.05
History of bleeding disorder	0.56	−0.76–1.89	0.4
Preoperative dialysis	2.87	−2.4–8.16	0.28
mFI-5	0.03	−0.42–0.49	0.87
ASA	0.81	0.17–1.45	0.013 ^¶^
Preoperative albumin level	−2.02	−2.63–−1.41	<0.001 ^¶^
Preoperative hematocrit level	−0.13	−0.2–−0.07	<0.001 ^¶^
Preoperative white cell count	0.1	0.03–0.18	0.003 ^¶^
Unplanned surgery	2.7	1.97–3.43	<0.001 ^¶^
Operative time	0.78	0.59–0.97	<0.001 ^¶^
Fusion procedure	0.21	−0.55–0.98	0.58
Perioperative transfusion	0.17	−0.75–1.09	0.71

^¶^ Independent predictor.

## Data Availability

All the data pertinent to this study are included in the article and Supplementary Materials. Further inquiries can be directed to the corresponding author.

## Supplementary Materials

The following supporting information can be downloaded at: https://www.mdpi.com/article/10.3390/cancers17142403/s1, Table S1. CPT codes for surgery for spinal metastases. Table S2. Univariable logistic regression of factors associated with failure to rescue for patients managed with metastatic spine tumor surgery for disseminated cancer. Table S3. Univariable logistic regression of factors associated with 30-day major (Clavien-Dindo Grade 3 or 4) complications for patients managed with metastatic spine tumor surgery for disseminated cancer. Table S4. Univariable logistic regression of factors associated with 30-day mortality for patients managed with metastatic spine tumor surgery for disseminated cancer. Table S5. Univariable linear regression of factors associated with length of hospital stay for patients managed with metastatic spine tumor surgery for disseminated cancer.
