# Supplementary material for: Short-Term Outcomes in Planned Versus Unplanned Surgery for Spinal Metastases
Source: Cancers (Basel). 2025 Jul 20;17(14):2403. doi: 10.3390/cancers17142403 (PMC12293508; doi:10.3390/cancers17142403)
Supplement: Supplementary file 1 [file cancers-17-02403-s001.zip › cancers-3746420-supplementary.pdf]

Supplementary Materials

Supplementary Table S1. CPT codes for surgery for spinal metastases

**Procedure**	**Codes**
Excision procedures on the spine	22100, 22101, 22102, 22013, 22110, 22112, 22114, 22116
Fracture and/or dislocation procedures on the spine (vertebral column)	22318, 22319, 22325, 22326, 22327, 22328
Transpedicular or costovertebral approach for posterolateral extradural exploration/decompression procedures on the spine and spinal cord	63055, 63056, 63057, 63064, 63066
Anterior or anterolateral approach for extradural exploration/decompression	63081, 63082, 63085, 63086, 63087, 63088, 63090, 63091

procedures on the spine and spinal cord (including vertebral corpectomy)	
Lateral extracavitary approach for extradural exploration/decompression procedures on the spine and spinal cord (including vertebral corpectomy)	63101, 63102, 63103
Excision by laminectomy of lesion other than herniated disk procedure	63275, 63276, 63277, 63278, 63280, 63281, 63282, 63283, 63285, 63286, 63287, 63290, 63295
Excision, anterior or anterolateral approach, intraspinal lesion procedures on the spine and spinal cord (including vertebral corpectomy)	63300, 63301, 63302, 63303, 63304, 63305, 63306, 63307, 63308

Supplementary Table S2. Univariable logistic regression of factors associated with failure to rescue for patients managed with metastatic spine tumor surgery for disseminated cancer

**Parameters**	**Odds Ratio**	**95% CI**	**p-value**
Age	1.01	0.99 – 1.03	0.25
Male sex	1.76	1.07 – 2.9	0.026*
Preoperative functional health status			
Independent		REFERENCE	
Partially dependent	2.44	1.33 – 4.45	0.004*
Totally dependent	3.66	1.07 – 12.46	0.038*
Unknown status	5.08	0.6 – 42.9	0.13
BMI	0.98	0.95 – 1.02	0.53
History of COPD	0.88	0.27 – 2.87	0.84
History of congestive heart failure	2.97	1.14 – 7.69	0.025*
History of bleeding disorder	3.1	1.7 – 5.68	< 0.001*
Preoperative dialysis	3.77	0.45 – 31.05	0.21
mFI-5	1.45	1.14 – 1.85	0.002*

ASA	2.38	1.58 – 3.58	< 0.001*
Chronic steroid use	1.44	0.86 – 2.42	0.16
Preoperative albumin level	0.34	0.24 – 0.48	< 0.001*
Preoperative hematocrit level	0.91	0.87 – 0.94	< 0.001*
Preoperative white cell count	1.09	1.05 – 1.12	< 0.001*
Unplanned surgery	2.81	1.76 – 4.5	< 0.001*
Operative time	0.97	0.86 – 1.09	0.63
Fusion procedure	0.83	0.52 – 1.31	0.42
Corpectomy	0.79	0.47 – 1.3	0.35
Multilevel corpectomy	1.56	0.55 – 4.41	0.39
Perioperative transfusion	2.17	1.34 – 3.51	0.002*

* Included in the multivariable regression analysis

Supplementary Table S3. Univariable logistic regression of factors associated with 30-day major (Clavien-Dindo Grade 3 or 4) complications for patients managed with metastatic spine tumor surgery for disseminated cancer

**Parameters**	**Odds Ratio**	**95% CI**	**p-value**
Age	1	0.99 – 1.01	0.2
Male sex	1	0.8 – 1.25	0.94
Preoperative functional health status			
Independent		REFERENCE	
Partially dependent	1.46	1.02 – 2.08	0.036*
Totally dependent	2.59	1.18 – 5.67	0.017*
Unknown status	1.86	0.36 – 9.67	0.45
BMI	0.98	0.96 – 1	0.17
History of COPD	1.52	0.93 – 2.48	0.08
History of congestive heart failure	2.26	1.25 – 4.09	0.007*
History of bleeding disorder	1.46	0.98 – 2.16	0.05
Preoperative dialysis	2.66	0.63 – 11.2	0.18
mFI-5	1.39	1.23 – 1.58	< 0.001*

ASA	1.56	1.28 – 1.9	< 0.001*
Chronic steroid use	1.12	0.85 – 1.46	0.41
Preoperative albumin level	0.5	0.42 – 0.61	< 0.001*
Preoperative hematocrit level	0.95	0.93 – 0.96	< 0.001*
Preoperative white cell count	1.04	1.02 – 1.06	< 0.001*
Unplanned surgery	1.32	1.05 – 1.64	0.013*
Operative time	1.05	0.99 – 1.11	0.05
Fusion procedure	1.09	0.87 – 1.37	0.43
Corpectomy	1.05	0.83 – 1.32	0.66
Multilevel corpectomy	1.86	1.1 – 3.14	0.019*
Perioperative transfusion	2.01	1.56 – 2.58	< 0.001*

* Included in the multivariable regression analysis

Supplementary Table S4. Univariable logistic regression of factors associated with 30-day mortality for patients managed with metastatic spine tumor surgery for disseminated cancer

**Parameters**	**Odds Ratio**	**95% CI**	**p-value**
Age	1.01	1 – 1.02	0.037*
Male sex	1.56	1.1 – 2.22	0.012*
Preoperative functional health status			
Independent		REFERENCE	
Partially dependent	2.04	1.28 – 3.24	0.003*
Totally dependent	2.33	0.79 – 6.83	0.12
Unknown status	2.33	0.27 – 19.56	0.43
BMI	0.97	0.94 – 1	0.06
History of COPD	1.23	0.58 – 2.59	0.58
History of congestive heart failure	2.82	1.34 – 5.9	0.006*
History of bleeding disorder	3.2	2.03 – 5.04	< 0.001*
Preoperative dialysis	4.26	0.85 – 21.31	0.07
mFI-5	1.36	1.14 – 1.63	0.001*

ASA	1.83	1.37 – 2.46	< 0.001*
Chronic steroid use	1.37	0.93 – 2.01	0.1
Preoperative albumin level	0.36	0.28 – 0.47	< 0.001*
Preoperative hematocrit level	0.91	0.89 – 0.94	< 0.001*
Preoperative white cell count	1.08	1.06 – 1.11	< 0.001*
Unplanned surgery	2.66	1.9 – 3.72	< 0.001*
Operative time	0.88	0.8 – 0.97	0.012*
Fusion procedure	0.61	0.44 – 0.85	0.004*
Corpectomy	0.84	0.59 – 1.2	0.35
Multilevel corpectomy	1.15	0.49 – 2.71	0.73
Perioperative transfusion	1.8	1.25 – 2.6	0.001*

* Included in the multivariable regression analysis

Supplementary Table S5. Univariable linear regression of factors associated with length of hospital stay for patients managed with metastatic spine tumor surgery for disseminated cancer

**Parameters**	**β**	**95% CI**	**p-value**
Age	0.02	- 0.002 – 0.05	0.07
Male sex	0.34	- 0.31 – 1.01	0.3
Preoperative functional health status			
Independent		REFERENCE	
Partially dependent	2.5	1.36 – 3.64	< 0.001*
Totally dependent	4.86	1.85 – 7.88	0.002*
Unknown status	7.1	1.54 – 12.65	0.012*
BMI	- 0.009	- 0.06 – 0.04	0.752
History of COPD	- 0.01	- 1.65 – 1.62	0.98
History of congestive heart failure	0.72	- 1.37 – 2.81	0.5
History of bleeding disorder	2.75	1.46 – 4.03	< 0.001*
Preoperative dialysis	5.81	0.22 – 11.4	0.04*
mFI-5	0.8	0.41 – 1.19	< 0.001*

ASA	2.21	1.64 – 2.78	< 0.001*
Chronic steroid use	- 0.45	- 1.28 – 0.37	0.28
Preoperative albumin level	- 3.17	- 3.73 – -2.61	< 0.001*
Preoperative hematocrit level	- 0.23	- 0.29 – - 0.18	< 0.001*
Preoperative white cell count	0.19	0.12 – 0.26	< 0.001*
Unplanned surgery	2.88	2.22 – 3.54	< 0.001*
Operative time	0.69	0.52 – 0.85	< 0.001*
Fusion procedure	1.14	0.46 – 1.82	0.001*
Corpectomy	0.66	- 0.03 – 1.36	0.06
Multilevel corpectomy	1.4	- 0.43 – 3.23	0.13
Perioperative transfusion	2.23	1.39 – 3.07	< 0.001*

* Included in the multivariable regression analysis
